# Indications, Clinical Outcomes, and Survival Rate of Pediatric Penetrating Keratoplasty in Suburban Malaysia: A 10-year Experience

**DOI:** 10.7759/cureus.3744

**Published:** 2018-12-17

**Authors:** Lam Mun-Wei, Haslinda Md Said, Rajendran Punitan, Mohtar Ibrahim, Ismail Shatriah

**Affiliations:** 1 Ophthalmology, School of Medical Sciences, Universiti Sains Malaysia, Kubang Kerian, MYS

**Keywords:** clinical outcome, pediatric penetrating keratoplasty, suburban malaysia, graft survival rate

## Abstract

Purpose

To describe the demographics, indications, clinical outcomes and survival rate of penetrating keratoplasty in Malaysian children living in a suburban area, and discuss the literature on paediatric penetrating keratoplasty.

Methodology

A retrospective review of medical records was performed on children younger than 17 years of age who had undergone penetrating keratoplasty in Hospital Universiti Sains Malaysia from January 2008 to December 2017. We recorded demographic data, presenting visual acuity, indications, final visual acuity, and graft survival at 12 months into the postoperative period.

Results

Sixteen eyes of 14 children had penetrating keratoplasty. Mean age was 7.8 ± 5.9 years. Both genders were equally affected. The main indications were infective keratitis (56.25%), congenital corneal opacity (18.75%) and trauma (12.50%). There were 62.50% of patients who had a preoperative visual acuity worse than 6/60. Fifty percent had other combined procedures during the surgery, including lens aspiration, peripheral iridectomy, pupilloplasty and glaucoma tube implant. Best corrected visual acuity of 6/12 or better was achieved in 18.75% of patients. A hazy graft was noted in 68.75% of patients, and was attributed to graft rejection, glaucoma and graft failure. There was a statistically significant association between the presence of vascularized cornea, intraocular inflammation and combined surgery with survival rate of the graft at one-year postoperative period (p < 0.05).

Conclusions

Infective keratitis is the main indication for penetrating keratoplasty in our pediatric patients. Good visual outcome was documented in a small percentage of the patients. Amblyopia and hazy graft were the main barriers to success in this group of patients. Vascularized cornea, inflammation and combined surgery had significantly affected the survival rate of the grafts in our series.

## Introduction

Pediatric keratoplasty is a challenging surgical procedure. Indications for pediatric keratoplasty differ between developing and developed countries. Trauma and infection are the most common causes of pediatric keratoplasty in developing countries [1-4]. However, keratoconus and congenital corneal opacities are the main reasons for pediatric keratoplasty in developed countries [5-10].

Available data on pediatric keratoplasty in Asian countries are limited. Published reports are mainly from India and China [1-2,11]. Recently, Low et al. described the primary outcome of pediatric keratoplasty in Singapore [10]. We aim to describe demographic data, indications and clinical outcomes of pediatric keratoplasty in Malaysia, and discuss the published literatures from both developing and developed countries.

## Materials and methods

A retrospective review was performed on 14 children younger than 17 years who underwent penetrating keratoplasty surgery in Hospital Universiti Sains Malaysia, Malaysia from January 2008 to December 2017. The patients were monitored for a minimum of three years period (range 36 to 96 months). This study was conducted according to the Declaration of Helsinki.

Hospital Universiti Sains Malaysia is a tertiary hospital on the east coast of Peninsular Malaysia. It serves as a referral centre and is equipped with cornea and pediatric ophthalmology subspecialty services. It receives referrals from the east coast states of Peninsular Malaysia, i.e. Kelantan, Terengganu and Pahang which cover an area of approximately 63,974 km^2^ with a population of 4.6 million people in 2018.

Patients less than 17 years of age who had undergone penetrating keratoplasty were included in our case review. All patients were co-managed by the cornea and pediatric ophthalmology consultants, during pre- and postoperative visits. Penetrating keratoplasty was performed by a single corneal surgeon. The glaucoma tube implant was done by a glaucoma consultant.

We recorded the patients' age during the initial presentation, gender, visual acuity before the procedure, indications, and period from diagnosis to the surgery. Other combined surgical procedures during the penetrating keratoplasty, culture and histopathological analysis of the recipient cornea, visual acuity at one year after the surgery, graft survival, and causes of poor final visual acuity were also reviewed and documented. Data was analysed using the Statistical Package for Social Sciences (SPSS) version 22 (IBM, Armonk, NY).

## Results

Sixteen penetrating keratoplasty procedures were performed in Hospital Universiti Sains Malaysia during the 10-year period. They were from 16 eyes of 14 children; one patient had procedures done in both eyes for congenital cornea opacity, while another patient undergone re-graft. The ages during the penetrating keratoplasty procedures ranged from 1 to 16 years (mean: 7.8 ± 5.9 years).

Nine eyes (56.25%) were operated on when the patients were less than five years of age, while six eyes (37.50%) had surgery when the patients were between 11 and 16 years. No gender predilection was observed. Right and left eyes were equally affected. Infective keratitis was documented in nine eyes (56.25%), followed by congenital corneal opacity in two eyes (12.50%), and trauma in two eyes (12.50%). These are presented in Table 1.

**Table 1:** Demographic characteristics and clinical diagnosis. ^a ^Calculated based on 14 patients.

Characteristics	No. (%)
Age range (years)	
0-5	9 (56.25)
6-10	1 (6.25)
11-17	6 (37.50)
Gender^a^	
Male	7 (50.00)
Female	7 (50.00)
Laterality	
Right eye	8 (50.00)
Left eye	8 (50.00)
Indication for penetrating keratoplasty	
Acquired traumatic	
Traumatic corneal scar	2 (12.50)
Acquired non-traumatic	
Perforated bacterial keratitis	3 (18.75)
Scarred bacterial keratitis	1 (6.25)
Herpetic keratitis	2 (12.50)
Interstitial keratitis	3 (18.75)
Corneal opacity secondary to Steven Johnson	1 (6.25)
Re-graft	1 (6.25)
Congenital	
Congenital cornea opacity	2 (12.50)
Bullous keratopathy secondary to congenital glaucoma	1 (6.25)

Ten eyes (62.50%) had visual acuity worse than 6/60 before the penetrating keratoplasty procedure, while six eyes (37.50%) were unable to be examined. No eyes displayed a visual acuity better than 6/60 before the procedure. Eight eyes (50.00%) had other combined procedures during the penetrating keratoplasty surgery. These procedures included lens aspiration (four eyes, 25.00%), peripheral iridectomy (two eyes, 12.50%), pupilloplasty and glaucoma tube implant (one eye, 6.25% for both). Nine eyes (56.25%) were operated on within one year of the initial consultation and diagnosis while the remaining patients took longer than a year due to poor socioeconomic status, refusal for surgery during the early phase and being unfit for general anesthesia when listed for surgery. The above information is summarized in Table 2.

**Table 2:** Clinical and surgical characteristics.

Characteristics	No. (%)
Visual acuity before procedure	
6/12 and better	0 (0.00)
6/15-6/60	0 (0.00)
Worse than 6/60	10 (62.50)
Not able to examine	6 (37.50)
Postoperative visual acuity at one year	
6/12 and better	3 (18.75)
6/15-6/60	1 (6.25)
Worse than 6/60	6 (37.50)
Unable to determine	6 (37.50)
Surgical procedure with penetrating keratoplasty	
Penetrating keratoplasty only	8 (50.00)
Combined with other procedures	
Lens aspiration	4 (25.00)
Peripheral iridectomy	2 (12.50)
Pupilloplasty	1 (6.25)
Molteno tube implant	1 (6.25)
Time from diagnosis to penetrating keratoplasty procedure	
Two weeks	3 (18.75)
Six months	2 (12.50)
One year	4 (25.00)
Two years	4 (25.00)
Three years	2 (12.50)
More than three years	1 (6.25)

We encountered great difficulty in obtaining samples for culture in infective cases. Four eyes (25.00%) were proven to be culture negative. Histopathological analyses revealed evidence of acute inflammation, acute suppurative keratitis, corneal scars, fibrosis and vascularization in four eyes (one eye per condition, 6.25%). The remaining results were unavailable.

Three eyes (18.75%) had a final visual acuity better than 6/12 at one year after the procedure. One eye (6.25%) achieved a best corrected visual acuity of 6/15 only due to amblyopia. Final visual acuities worse than 6/60 were documented in six eyes (37.50%), while visual acuities were not able to be assessed in the remaining six eyes (37.50%). Out of 12 eyes with poor final visual acuity, hazy grafts were noted in 11 eyes (68.75%). These were due to graft rejections in seven eyes (43.75%) which mostly contributed by infective cases, glaucoma in three eyes (18.75%), and a graft failure in one eye (6.25%). The relevant data are presented in Table 3.

**Table 3:** Survival of the graft at one year.

Characteristics	No. (%)
Clear graft	5 (31.25)
Hazy graft	
Postoperative rejection	7 (43.75)
Postoperative glaucoma	3 (18.75)
Graft failure	1 (6.25)

**Figure 1:**
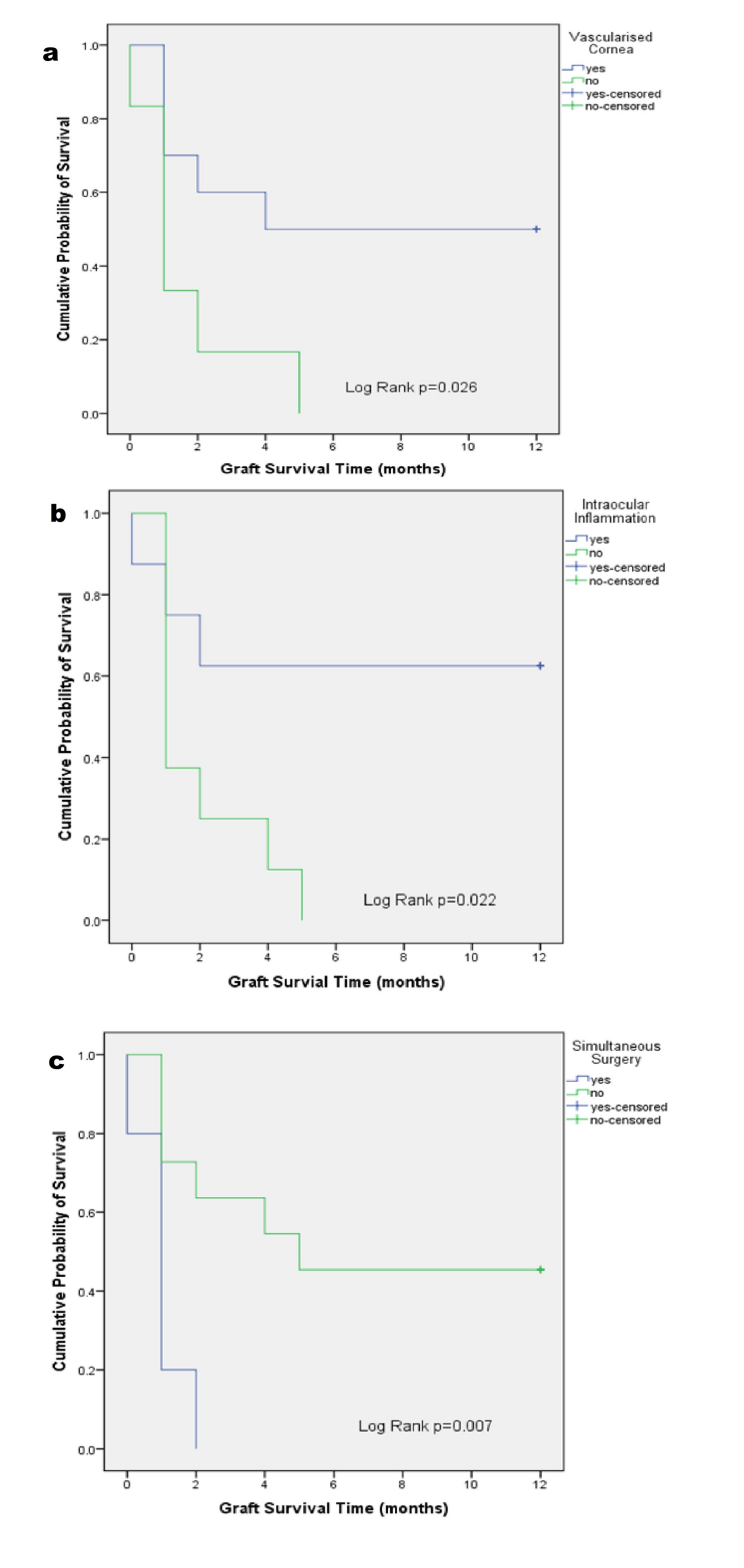
The Kaplan-Meier graft survival for (a) grafts with vascularized cornea, (b) grafts with intraocular inflammation and (c) grafts undergoing simultaneous surgery.

The Kaplan-Meier survival rates showed significant association between the risk factors such as vascularized cornea (p = 0.026), intraocular inflammation (p = 0.022) and simultaneous surgery (p = 0.007) with the survival of corneal graft (Figure 1). Factors like previous surgery performed, glaucoma, iridocorneal touch, re-graft and age were analyzed but showed no significant association with the outcome of corneal graft survival rate.

## Discussion

The majority of pediatric keratoplasty reviews have described penetrating keratoplasty as the main procedure involved [1-11]. Other procedures, such as anterior lamella keratoplasty and Descemet-stripping automated endothelial keratoplasty have also been described in a few reports [2,5,10-13]. Table 4 summarizes published studies on pediatric keratoplasty from China, United States, New Zealand, Singapore, India, Finland, Denmark, Tunisia, Saudi Arabia, Australia and our present study [2,4-5,10-16].

**Table 4:** Published data on pediatric keratoplasty from 2003 till 2018. CHED: Congenital hereditary endothelial dystrophy; MPS: Mucopolysaccharidosis; NA: Not available.

Variables	Present Study	Kusumesh et al. [11]	Shi et al. [2]	Low et al. [10]	Al-Ghamdi et al. [15]	Patel et al. [5]	McClellan et al. [16]	Limaiem et al. [14]	Hovlykke et al. [13]
Country/Year	Malaysia/2018	India/2015	North China/2007	Singapore/2014	Saudi Arabia/2007	New Zealand/2005	Australia/2003	Tunisia/2011	Denmark/2013
Number of eyes	16	66	410	44	165	58	19	16	65
Number of patients	14	66	371	44	134	52	16	15	60
Range (years)	1–17	2 months–12	2.5 months–14	1 month–15	5 months–12	2 weeks–14	2 weeks–15	3 months-14	<16
Mean age (years)	7.8	4.0	7.8	8.4	NA	10.6	9.2	11.2	NA
Gender	M=F 7:7	M>F 43:23	M>F 259:112	M>F 67:38	NA	M>F 34:18	M>F 10:6	M>F 10:5	NA
Indication for Graft, n (%)									
1. Congenital	3 (18.75)	24 (36.36)	112 (27.32)	18 (40.90)	130 (78.79)	9 (15.52)	8 (42.11)	2 (12.5)	3 (4.62)
CHED	NA	6 (9.09)	NA	1 (2.27)	35 (21.21)	NA	1 (5.26)	NA	NA
Non-CHED	NA	6 (9.09)	NA	NA	NA	NA	NA	NA	NA
Anterior segment dysgenesis	NA	6 (9.09)	NA	15 (34.09)	28 (16.97)	4 (6.90)	3 (15.79)	NA	NA
Aniridia	NA	NA	NA	NA	NA	2 (3.45)	2 (10.53)	NA	2 (3.08)
MPS Type 1	NA	NA	NA	NA	NA	NA	1 (5.26)	NA	NA
Cornea dystrophy	NA	NA	29 (7.07)	NA	NA	NA	NA	1 (6.25)	NA
Cornea opacity	2 (12.50)	6 (9.09)	53 (12.93)	NA	NA	2 (3.45)	1 (5.26)	1 (6.25)	NA
Congenital glaucoma	1 (6.25)	NA	NA	1 (2.27)	49 (29.70)	NA	NA	NA	NA
Limbal dermoid	NA	NA	30 (7.32)	1 (2.27)	6 (3.64)	1 (1.72)	NA	NA	NA
Others	NA	NA	NA	NA	12 (7.27)	NA	NA	NA	1 (1.54)
2. Acquired non-traumatic, n (%)	11 (68.75)	37 (56.06)	148 (36.10)	26 (59.10)	18 (10.91)	43 (74.14)	11 (57.89)	8 (50.00)	54 (83.08)
Keratoconus	NA	NA	37 (9.02)	10 (22.73)	NA	39 (67.24)	8 (42.11)	5 (31.25)	12 (18.46)
Infectious keratitis	9 (56.25)	22 (33.33)	93 (22.68)	4 (9.09)	18 (10.91)	4 (6.90)	1 (5.26)	3 (18.75)	20 (30.77)
Cornea perforation	NA	NA	NA	2 (4.55)	NA	NA	NA	NA	NA
Steven Johnson Syndrome	1 (6.25)	NA	NA	NA	NA	NA	NA	NA	NA
Adherent leukoma	NA	NA	NA	NA	NA	NA	1 (5.26)	NA	NA
Keratomalacia	NA	11 (16.67)	7 (1.71)	NA	NA	NA	NA	NA	NA
Regraft	1 (6.25)	4 (6.06)	NA	NA	NA	NA	1 (5.26)	NA	12 (18.46)
Bullous keratopathy	NA	NA	NA	1 (2.27)	NA	NA	NA	NA	NA
Cornea scar	NA	NA	NA	8 (18.18)	NA	NA	NA	NA	NA
Iris tumor	NA	NA	NA	1 (2.27)	NA	NA	NA	NA	NA
Unknown	NA	NA	11 (2.68)	NA	NA	NA	NA	NA	6 (9.23)
Others	NA	NA	NA	NA	NA	NA	NA	NA	4 (6.15)
3. Acquired traumatic	2 (12.5)	5 (7.58)	150 (36.6)	0 (0.00)	17 (10.30)	6 (10.34)	NA	6 (37.50)	8 (12.31)
Visual acuity at one year, n (%)									
6/12 or better	3 (18.75)	NA	NA	NA	8 (4.80)	18 (35.29)	7 (36.8)	2 (12.50)	NA
6/15 to 6/60	1 (6.25)	NA	NA	NA	52 (31.51)	16 (32.00)	3 (15.8)	6 (37.50)	NA
Worse than 6/60	6 (37.5)	NA	NA	NA	105 (63.63)	12 (25.32)	9 (47.4)	8 (50.00)	NA
Not able to examine	6 (37.5)	NA	NA	NA	NA	5 (9.80)	NA	NA	NA
Graft survival at one year, n (%)									
Clear graft	5 (31.75)	NA	NA	(92.80)	73 (44.24)	42 (82.00)	14 (73.68)	8 (52.00)	38 (60.00)
Graft rejection	7 (47.35)	NA	NA	NA	NA	NA	1 (5.26)	4 (24.00)	NA
Glaucoma	3 (18.75)	NA	NA	NA	NA	NA	NA	NA	NA
Graft failure	1 (6.25)	NA	NA	NA	92 (55.75)	8 (16.00)	3 (15.70)	NA	25 (40.00)
Secondary trauma	NA	NA	NA	NA	NA	NA	1 (5.26)	3 (19.00)	NA
Inflammation	NA	NA	NA	NA	NA	NA	NA	1 (5.00)	NA
Died	NA	NA	NA	NA	NA	1 (5.00)	NA	NA	NA

The mean age in our study is consistent with a report by Shi et al., in 2007 who conducted a large retrospective study in North China (7.8 ± 4.3 years) [2]. Low et al. reported mean age of 8.4 ± 5.63 years in their review [10]. In contrast, a relatively younger age group was observed by Kusumesh and Vanathi, from India [11]. The mean age reported from developed countries, ranging from 9.2 to 12 years, is slightly older than those reported from developing countries [5,12-16].

We found no gender predilection in our present cases of pediatric keratoplasty. Similar observations have also been reported by Huang et al. and Dana et al., where both genders were equally affected [4,17]. On the other hand, a majority of the literature from Asia, North Africa, Australia and the United States have observed that boys outnumbered girls in their studies on pediatric keratoplasty [2,10-11,14,16,18].

The indication of penetrating keratoplasty is broadly classified into congenital, acquired non‑traumatic, and acquired traumatic corneal opacities. Our results showed that the most common indication was infective keratitis (56.25%), which belongs to the acquired non-traumatic group. Infective keratitis has been reported to be the leading cause in India (33.33%) and Denmark (30.77%) [11,13]. Keratoconus is the most common indication of pediatric keratoplasty in New Zealand (67.26%) and Australia (42.11%) [5,16]. Congenital corneal opacity is the main indication in the United States (61.60%), Singapore (40.90%) and Saudi Arabia (78.79%) [4,10,15].

Corneal lacerations due to trauma (12.50%) were the second highest in our series. Trauma was reported as the most common cause of pediatric keratoplasty in North China (36.60%), Finland (25.0%) and Tunisia (37.5%) [2,12,14]. Majander et al. mentioned that they had a higher percentage of keratoplasty indicated for traumatic corneal scars, which were performed to facilitate other intraocular surgeries. On the other hand, no penetrating keratoplasty secondary to corneal trauma was reported from Singapore and Australia [10,16].

Our series documented a final visual acuity of 6/12 or better in only 18.75% (three eyes). This data is consistent with a report by Limaiem et al., who reported that 12.20% of their patients had satisfactory final visual acuity, and ocular trauma contributed to 37.50% in their study [14]. On the contrary, Patel et al. and McClellan et al. observed more encouraging visual acuity results in their reports (35.29%, 36.8% respectively) [5,16]. Most of their patients had undergone penetrating keratoplasty due to keratoconus, which carries a better prognosis.

Al-Ghamdi et al. from Saudi Arabia reported that more than half of their patients achieved a final visual acuity worse than 6/60, and they attributed this finding to a majority of their patients having congenital corneal opacities [15]. Congenital corneal abnormalities are difficult to manage as they are usually associated with concurrent ocular diseases and amblyopia, which lead to poor visual outcomes [15,17]. However, there was no data available on final visual acuities from North China, the United States, Singapore, India, Finland and Denmark [2,4,10-13].

Our study demonstrated that only 31.25% (five eyes) of the patients had clear grafts after one year following the procedure. Other published reports documented higher percentage of patients with clear graft (range from 52 to 90%) [4,5,13-14,16]. Hazy cornea observed in the majority of our patients was due to postoperative rejections (43.75%), persistent glaucoma (18.75%), and graft failure (6.25%). Refusal and poor compliance to amblyopia treatment were also identified in our patients. On the other hand, a lower percentage of postoperative rejections was observed in Tunisia (24.0%) and Australia (5.26%) [14,16]. Graft failure was noted in the reports by Patel et al. (16.0%), Hovlykke et al. (40.0%), Al-Ghamdi et al. (55.75%), and McCellan et al. (15.6%). However, graft clarity was not mentioned in the studies performed in North China, India and Finland [2,11-12].

All our patients who had combined procedures (eight eyes) developed poor final visual acuity. Our observation is consistent with other authors who reported poor graft survivals in cases with combined surgical procedures [5,9,10,19,20]. Kusumesh et al. reported that two of their eight graft rejections occurred in cases where penetrating keratoplasty was performed as a combined procedure [11]. Patel et al. and Low et al. concurred that combined intraoperative procedures are a risk factor for graft failure [5,10]. Likewise, Al-Ghamdi et al. observed that combined glaucoma (graft survival 28%) and cataract (graft survival 19.2%) surgeries reduce the rate of graft survival [15]. Several risk factors are known to affect long-term corneal graft survival. In our study, vascularized corneal, simultaneous surgery and intraocular inflammation affected graft survival rate (Figure 1). These risk factors were consistent with other reported studies [5,10,12,13]. Other factors that were known to affect graft survival rates such as previous surgery performed, glaucoma, re-graft and iridocorneal touch were not significantly associated with our graft survival rates. This is possibly due to the limited small sample size collected in our retrospective study.

## Conclusions

Infective keratitis is the main indication for pediatric keratoplasty in our series. Both genders were equally affected. Less than one-third of the patients had clear grafts. A majority had poor visual acuity due to amblyopia and hazy grafts that resulted from graft rejections, persistent glaucoma, and graft failures. Our findings support the existing data regarding paediatric keratoplasty from developing countries. More workup and efforts are necessary to improve the diagnosis, treatment, and postoperative care for children who require keratoplasty from developing countries.
